# CD38/cADPR‐mediated calcium signaling in a human myometrial smooth muscle cell line, PHM1

**DOI:** 10.1002/iub.2904

**Published:** 2024-08-12

**Authors:** Soner Dogan, Timothy F. Walseth, Bilge Guvenc Tuna, Eda Uçar, Mathur S. Kannan, Deepak A. Deshpande

**Affiliations:** ^1^ Department of Medical Biology, School of Medicine Yeditepe University Istanbul Turkey; ^2^ Department of Veterinary and Biomedical Sciences University of Minnesota St. Paul Minnesota USA; ^3^ Department of Pharmacology University of Minnesota Minneapolis Minnesota USA; ^4^ Department of Biophysics, School of Medicine Yeditepe University Istanbul Turkey; ^5^ Center for Translational Medicine, Jane and Leonard Korman Lung Center Thomas Jefferson University Philadelphia Pennsylvania USA

**Keywords:** cADPR, CD38, endothelin‐1 (ET‐1), hormone, intracellular calcium, myometrium, oxytocin, prostaglandin F2α (PGF2α), smooth muscle, uterus

## Abstract

Cyclic ADP‐ribose (cADPR) has emerged as a calcium‐regulating second messenger in smooth muscle cells. CD38 protein possesses ADP‐ribosyl cyclase and cADPR hydrolase activities and mediates cADPR synthesis and degradation. We have previously shown that CD38 expression is regulated by estrogen and progesterone in the myometrium. Considering hormonal regulation in gestation, the objective of the present study was to determine the role of CD38/cADPR signaling in the regulation of intracellular calcium upon contractile agonist stimulation using immortalized pregnant human myometrial (PHM1) cells. Western blot, immunofluorescence, and biochemical studies confirmed CD38 expression and the presence of ADP‐ribosyl cyclase (2.6 ± 0.1 pmol/mg) and cADPR hydrolase (26.8 ± 6.8 nmoles/mg/h) activities on the PHM1 cell membrane. Oxytocin, PGF_2α_, and ET‐1 elicited [Ca^2+^]_i_ responses, and 8‐Br‐cADPR, a cADPR antagonist significantly attenuated agonist‐induced [Ca^2+^]_i_ responses between 20% and 46% in average. The findings suggest that uterine contractile agonists mediate their effects in part through CD38/cADPR signaling to increase [Ca^2+^]_i_ and presumably uterine contraction. As studies in humans are limited by the availability of myometrium from healthy donors, PHM1 cells form an in vitro model to study human myometrium.

## 1 INTRODUCTION

CD38 is a transmembrane protein with a catalytic site usually located extracellularly (ectoenzyme), although it could also be located intracellularly.^1^ Biochemically, CD38 is a bifunctional enzyme that synthesizes cyclic ADP‐ribose (cADPR) from β‐NAD^+^ by its ADP‐ribosyl cyclase activity and ADPR from cADPR or NAD by its hydrolase activity.^2^, ^3^ Besides synthesis of cADPR and ADPR, in acidic conditions, CD38 has also been demonstrated to form nicotinic acid adenine dinucleotide 2′‐phosphate (NAADP), which is reported to be the most potent second messenger for calcium release in the literature up to date.^4^ In this context, CD38, by forming cADPR majorly, releases calcium via IP_3_‐independent pathway from intracellular calcium stores.^5^

CD38 is expressed in multiple tissues such as endothelial cells,^6^ pancreas,^7^, ^8^, ^9^ liver,^10^ brain,^11^ skeletal muscle,^12^ heart muscle,^13^ and smooth muscle.^14^, ^15^ In this context, several studies have investigated the roles of CD38 activities for the intracellular calcium release in the myometrium.^16^, ^17^, ^18^, ^19^, ^20^, ^21^ For example, the contribution of cADPR‐mediated calcium release during oxytocin stimulation has been reported both in the human myometrial cells obtained from patients undergoing elective hysterectomy^17^ and in murine myometrial cells.^18^ Moreover, complete inhibition of either cADPR agonist or TNF‐alpha‐induced calcium release from intracellular stores by progesterone in human myometrial cells obtained with elective hysterectomy has also been reported.^18^ On the contrary, our group and others have demonstrated the expression of CD38 and its enzyme activities in the uterine smooth muscle with changing hormonal levels like estrogen^22^ and progesterone.^18^, ^23^ Specifically, we demonstrated increased levels of CD38 mRNA and protein expression in term‐pregnant rats compared with preterm ones. This increment was significantly associated with increased ADP‐ribosyl cyclase activity, resulting in significantly higher levels of cADPR in term rats compared with preterm ones.^23^ Collectively, these results indicate a hormonal regulation of CD38 in myometrium. Therefore, it is quite important to explore the roles of CD38 and its enzyme activities, which results in cADPR and ADPR production in calcium signaling during the gestational period. There are various studies using primary human myometrial cells that are received from an elective hysterectomy.^16^, ^17^, ^18^, ^19^, ^20^ However, to our knowledge, this paper is the first study to investigate CD38 expression and calcium responses to cADPR agonists (oxytocin, PGF‐2, and ET‐1) and antagonist 8‐Br‐cADPR in a gestational human myometrial tissue. For this purpose, pregnant human myometrium (PHM1) cells that are obtained from a term‐pregnant patient and immortalized with papillomavirus are used.

## 2 MATERIALS AND METHODS

### 2.1 Materials

Tris base, Tris–HCl, glucose, HEPES, nicotinamide guanine dinucleotide (NGD), cyclic guanosine diphosphoribose (cGDPR), 1,1,2,‐trichlorotrifluoroethane, tri‐n‐octylamine, oxytocin, PGF_2α_, ET‐1, and other chemicals were purchased from Sigma chemical company (St. Louis, MO). Hanks Balanced Salt Solution (HBSS), hexamers, oligo(dT), Taq DNA polymerase, and 100 bp DNA ladder were purchased from GibcoBRL (Grand Island, NY). Cellulose PEI TLC plates were purchased from Fisher Scientific (Pittsburgh, PA). Mouse anti‐human CD38 antibody (Santa Cruz Biotechnology, sc‐7325) (Santa Cruz), donkey anti‐goat IgG, and horseradish peroxidase were purchased from Santa Cruz Biotechnology (Santa Cruz, CA). Gradient gels and a Bio‐Rad protein assay kit were purchased from Bio‐Rad Laboratories (Hercules, CA). Protease inhibitor cocktail set III (Cat. No. 539134) was obtained from Calbiochem (La Jolla, CA). RNeasy mini kit was obtained from Qiagen (Valencia, CA). Resazurin, diaphorase, nicotinamide, nucleotide pyrophosphatase from Crotalus atrox venom, NADase from Neurospora crassa, and alkaline phosphatase from calf intestine were purchased from Boehringer. Cy3‐conjugated anti‐mouse IgG was obtained from Chemicon (Temecula, CA). Fura‐2/AM was purchased from molecular probes (Eugene, OR). 8‐Br‐cADPR was synthesized as described previously.^24^

### 2.2 Cell culture

In this study, immortalized PHM1 cells maintained in culture were used. These cells were obtained from Dr. Barbara M. Sanborn, Department of Biomedical Sciences, Colorado State University, Fort Collins, CO. PHM1 cells were isolated from the myometrium of a term‐pregnant woman and immortalized using a vector expressing human papillomavirus E6 and E7 proteins as described previously.^25^ There were five passages used in the experiments (p8‐p13). As it was explained in earlier studies, immortalized pregnant human myometrial cells (PHM1‐31 and PHM1‐41) retain many morphological and phenotypic responses of primary myometrial cells, express smooth muscle‐specific a‐actin, and retain oxytocin receptors.^26^ The cells were cultured in Dulbecco's minimum essential medium (DMEM) supplemented with 10% fetal bovine serum, 100 U/mL of penicillin, and 0.1 mg/mL of streptomycin and 2 mM l‐glutamine in a humidified atmosphere of 5% CO_2_ at 37°C.

### 2.3 CD38 mRNA expression in PHM1 cells

CD38 mRNA expression in PHM1 cells was determined as described previously.^21^ Briefly, total RNA was extracted using the RNeasy mini kit from Qiagen (Valencia, CA) as per the manufacturer's protocol. Reverse transcription of total RNA to first‐strand cDNA was done using random hexamers and oligo (dT) primers. cDNA was amplified by PCR using human CD38‐specific primers. The PCR primers were designed using a nucleotide sequence for rat CD38 (gi 497839 Gene Bank accession number). The following primers were used in the study: sense‐5′TGCAACAAGATTCTTCTTTGGAGCA3′ (position between 400 and 425) and anti‐sense‐5′CTCAGGATTTTTCACACACTGAAG3′ (position between 876 and 900), giving a final product of 500 bp. Rat uterine sample which was shown to be CD38‐positive in our previous publications was used as the positive control sample.

### 2.4 Western blot analysis

CD38 protein expression in PHM1 cells was determined by western blot analysis as described previously.^21^ Briefly, PHM1 cells were grown to confluence in 100 mm petri dishes, media was removed, and then the cells were washed with HBSS. Subsequently, cells were scraped and collected in sucrose‐Tris solution containing 43 g sucrose (0.25 M), 1.58 g Tris–HCL (20 mM) completed to 500 mL with water and protease inhibitors added (200 μL for 100 mL solution, prepared in pH 7.2). Later, cells in sucrose‐Tris solution were homogenized using a sonicator. The total protein in the homogenized samples was measured using the Bio‐Rad protein assay. Proteins from PHM1 samples were separated on 4%–15% polyacrylamide gradient gels and subsequently transferred to a polyvinylidene difluoride membrane. The protein on the membrane was probed using a human anti‐CD38 antibody raised in mouse with a dilution at 1:100 (Santa Cruz Biotechnology, sc‐7325) as described in our previous publication.^27^ This antibody has been validated for the following applications: flow cytometry, immunofluorescence, immunohistochemistry—fixed, immunoprecipitation, and western blot. A horseradish peroxidase‐conjugated donkey anti‐mouse goat IgG was used as a secondary antibody. The blots were developed using the Bio‐Rad Chemiluminescence detecting system. Rat uterine samples which were shown to be CD38‐positive in our previous publications were used as positive control samples.

### 2.5 Immunofluorescence staining of CD38

PHM1 cells were grown on glass coverslips and fixed using 4% paraformaldehyde in phosphate‐buffered saline (pH 7.4) for 10 min. After washing the cells three times with PBS, the cells were exposed to 10% normal goat serum for 20 min. Subsequently, the cells were incubated with mouse anti‐human CD38 antibody (Santa Cruz Biotechnology, sc‐7325) with a dilution at 1:100 in 1.5% normal goat serum overnight at 4°C. The cells were washed with PBS five times and incubated with Cy3 conjugated goat anti‐mouse IgG for 1 h. The cells were washed with PBS 5 times and mounted onto a glass slide using SlowFade mounting medium (Molecular Probes, OR). The stained cells were visualized using fluorescence microscope. Cells stained with no primary antibody were used as the negative control.

### 2.6 ADP‐ribosyl cyclase activity in PHM1 cells

ADP‐ribosyl cyclase activity in PHM1 cells was assessed by measuring the conversion of NGD, an analog of nicotinamide adenine dinucleotide, to cGDPR using a spectrofluorometer (Shimadzu Corporation, Kyoto, Japan), as described earlier.^21^, ^28^ Briefly, PHM1 cells were grown to confluence in 100 mm petri dishes, cell media (DMEM) was removed, and the cells were washed with HBSS. Subsequently, cells were scraped and collected in sucrose‐Tris solution containing protease inhibitors. Later, cells in sucrose‐Tris solution were homogenized using a sonicator. The total protein in the homogenized samples was measured using the Bio‐Rad protein assay. The reaction was started by the addition of NGD (200 μM) to whole cell lysate in sucrose‐Tris and Tris–HCl mix solution. The specific ADP‐ribosyl cyclase activity was calculated using known cGDPR standards. The results were represented as nanomoles cGDPR formed/milligram protein/hour.

### 2.7 cADPR hydrolase activity in PHM1 cells

The cADPR hydrolase activity was measured in PHM1 cells using ^32^P‐cADPR as a substrate, as described earlier.^21^ Briefly, PHM1 cells homogenized in sucrose‐Tris solution containing protease inhibitors were incubated at 37°C in 20 mM Tris–HCl solution, pH 7.2 containing ^32^P‐cADPR (500 cpm/μL per each sample) and 600 μM unlabeled cADPR. At different time points, 1 μL of the reaction mixture was spotted on a PEI cellulose plate. The plate was developed in a solution containing 30% ethanol and 0.2 M NaCl, dried, and exposed to a phosphorimager screen. The amount of cADPR hydrolyzed to ADPR in the reaction was calculated by quantifying the densities of the spots corresponding to cADPR and ADPR using the Optiquant Imaging System. The cADPR hydrolase enzyme activity was expressed as nanomole ADPR/milligram protein/hour.

### 2.8 Determination of cADPR levels in PHM1 cells

Basal levels of cADPR in PHM1 cells grown in normal DMEM media were measured using a cycling assay as described previously.^28^ Briefly, after removing the medium, confluent PHM1 cells were scraped in 0.5 M ice‐cold perchloric acid (PCA) solution and homogenized in PCA solution. After removal of protein by centrifugation at 7000×*g* for 7 min, PCA was removed by mixing the supernatant with a mixture of 1,1,2,‐trichlorotrifluoroethane and tri‐n‐octylamine (3:1 volume). Following vigorous mixing for 30 s and centrifugation of the samples at 7000×*g* for 5 min, the upper aqueous layer was recovered and adjusted to pH 7.5 by adding Tris‐base (2–5 μL of 2 M). Subsequently, the samples were treated with enzymes to remove pyridine nucleotides and cADPR levels were assayed as described previously.^28^ Briefly, enzymes‐treated extracts from PHM1 cells were incubated with reagents containing ADP‐ribosyl cyclase, nicotinamide, ethanol, alcohol dehydrogenase, diaphorase, flavin mononucleotide, and resazurin. The increased fluorescence product of resazurin, called resorufin, was measured using a fluorometer.

### 2.9 Measurement of intracellular calcium concentration

Cell‐permeant, ratiometric calcium indicator dye, fura‐2/AM was used to determine the intracellular calcium concentration in PHM1 cells. PHM1 cells were grown in DMEM with 10% FBS on glass coverslips for 48 h. Cells were washed and maintained in HBSS containing 10 mM HEPES (pH 7.4), 11 mM glucose, 2.5 mM CaCl_2_, and 1.2 mM MgCl_2_ and incubated with 5 μM fura‐2/AM for 30 min at 37°C in 5% CO_2_. An open slide chamber was used to mount the coverslip, which was subsequently placed on the stage of a Nikon Diaphot inverted microscope. The cells were perfused with serum and HBSS, and the basal intracellular calcium concentration ([Ca^2+^]_i_) was determined using real‐time digital video fluorescence imaging (Metafluor: Universal Imaging Corporation) as described previously.^29^ Fura‐2/AM‐loaded cells were alternately excited at 340 and 380 nm using a Lambda DG‐4 high‐speed filter changer (Sutter Instrument Company). Fluorescence emissions were collected separately for each wavelength using a 510 nm barrier filter. Photometric Cool Snap 12‐bit digital camera (Roper Scientific) was used to collect images at the rate of one image for every 0.75 s, and ratios of fluorescence intensities at 340 and 380 nm were determined at each time point. 4‐Br‐A23187 was used to determine the maximum while EGTA was used to determine the minimum calcium concentration in PHM1 cells, and these images were used to obtain a calcium calibration curve, as described previously.^27^

Oxytocin, prostaglandin‐F_2α_ (PGF_2α_), and endothelin‐1 (ET‐1) were used as agonists. The basal [Ca^2+^]_i_ was determined by perfusing the fura‐2/AM‐loaded PHM1 cells with HBSS. Subsequently, the cells were perfused with HBSS containing different concentrations (10 nM, 100 nM, 1 μM) of the agonists (Oxytocin, PGF_2α_, or ET‐1) for at least 2–3 min. The net intracellular Ca^2+^ responses to agonists were calculated by subtracting basal [Ca^2+^]_i_ from the peak [Ca^2+^]_i_. All experiments were carried out at room temperature. Cells grown on a coverslip were exposed to only one of the three agonists. Experiments were conducted on at least two different days for each agonist (2–7 different days). For each concentration trial of each agonist, two to five replicates were done (or slides were imaged) on each day. A total of 4–12 experiments for each concentration of each agonist (*n* = 4–12) were performed. The experiments were conducted on different days with different badges of samples. For image analysis, a total of 70–412 cells were used.

### 2.10 Effect of 8‐Br‐cADPR on agonist‐induced intracellular Ca^2+^ response

To determine the contribution of cADPR to agonist‐elicited [Ca^2+^]_i_ responses, PHM1 cells were pre‐incubated with either HBSS or 100 μM of a membrane‐permeant cADPR antagonist, 8‐Br‐cADPR for 15 min. Subsequently, the cells were stimulated with different concentrations of contractile agonists, and the net [Ca^2+^]_i_ responses were determined as described above. In a different set of experiments, PHM1 cells were pre‐incubated with different concentrations (10 μM, 100 μM, 1 mM) of 8‐Br‐cADPR for 15 min. The basal [Ca^2+^]_i_ was determined by perfusing the fura‐2/AM‐loaded PHM1 cells with HBSS. Subsequently, the cells were perfused with HBSS containing 100 nM of oxytocin for 2–3 min. The net [Ca^2+^]_i_ responses to 100 nM oxytocin in control were compared with the net [Ca^2+^]_i_ responses in PHM1 cells treated with different concentrations of 8‐Br‐cADPR.

### 2.11 Statistical analysis

The statistical analysis was done using the GraphPad PRISM statistical software. One‐way ANOVA with Bonferroni's test for multiple comparisons was used for statistical analysis. Two means were considered significantly different when *p* value was less than .05, and data are presented as mean ± standard error of means (SEM).

## 3 RESULTS

### 3.1 PHM1 cells express CD38

**FIGURE 1:**
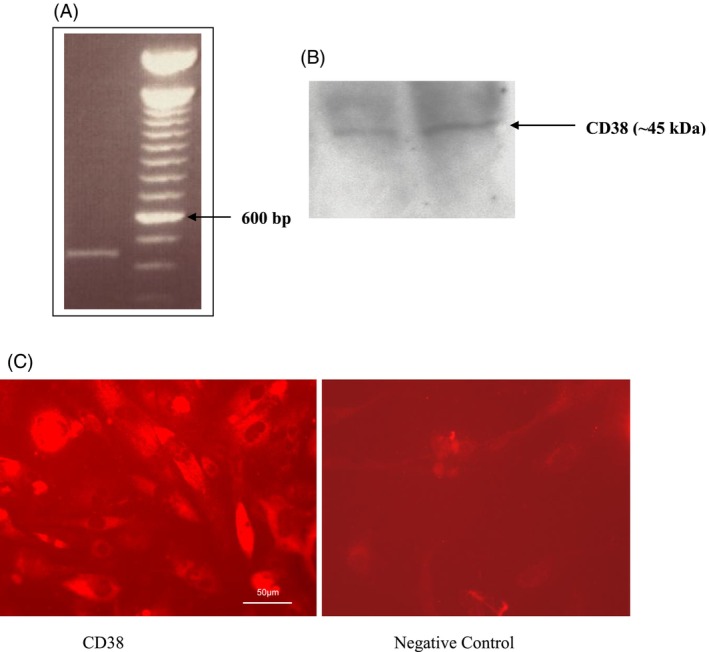
Detection of CD38 mRNA and protein expression in PHM1 cells. (A) Detection of CD38 mRNA expression in PHM1 cells by RT‐PCR (*n* = 4). (B) Detection of CD38 protein expression in PHM1 cells by western blot analysis using a goat‐polyclonal anti‐rat CD38 antibody. The data shown are a representative blot from one prep shown in duplicate that was performed using three different preparations (*n* = 3). (C) Detection of CD38 protein expression by immunofluorescence staining using mouse anti‐human CD38 antibody. Cells stained with no CD38 primary antibody or stained with IgG were used for negative control (*n* = 4).

CD38 expression in PHM1 cells was determined by RT‐PCR, western blot analysis, and immunofluorescence staining, as described in materials and methods. RT‐PCR using total RNA isolated from PHM1 cells revealed the expression of CD38 mRNA in PHM1 cells (Figure 1A). Furthermore, both the western blot analysis (Figure 1B) and immunofluorescence staining (Figure 1C) demonstrated the expression of CD38 protein in PHM1 cells.

### 3.2 CD38 enzyme activities and cADPR levels in PHM1 cells

**FIGURE 2:**
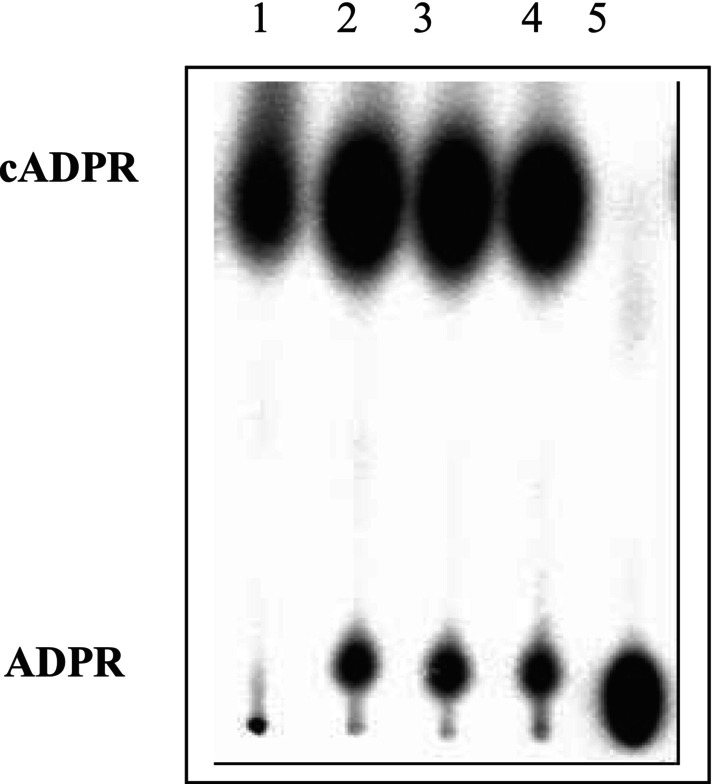
Detection of cADPR hydrolase enzyme activity in PHM1 cells. Lane 1: negative control (buffer was used instead of PHM1 cell whole lysate), Lane 2–4: PHM1 cell samples from three different preparations, Lane 5: positive control (rat heart microsomes).

ADP‐ribosyl cyclase activity in PHM1 cells was measured using the NGD assay as described in materials and methods. The basal ADP‐ribosyl cyclase activity was 7.7 ± 4.4 nmoles/mg/h (*n* = 2). cADPR hydrolase activity was determined using ^32^P‐cADPR as the substrate. The cADPR hydrolase activity in PHM1 cells was 26.8 ± 6.8 nmoles/mg/h (*n* = 3) (Figure 2). The basal level of cADPR, using the cycling assay, was 2.6 ± 0.1 pmol/mg protein in PHM1 cells (*n* = 3).

### 3.3 Agonist‐induced [Ca^2+^]_i_ increase in PHM1 cells

**FIGURE 3:**
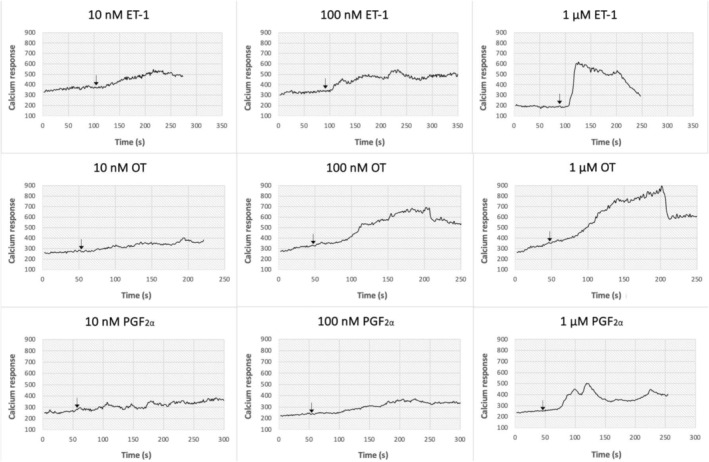
Intracellular calcium responses to different concentrations of different agonists in PHM1 cells. Cells were stimulated with 10 nM, 100 nM, and 1 μM of endothelin‐1 (top row), oxytocin (middle row), and PGF2α (bottom row). Levels of [Ca^2+^]_i_ increase in PHM1 cells were measured as explained in materials and methods. Arrows indicate when the agonists were added.

**FIGURE 4:**
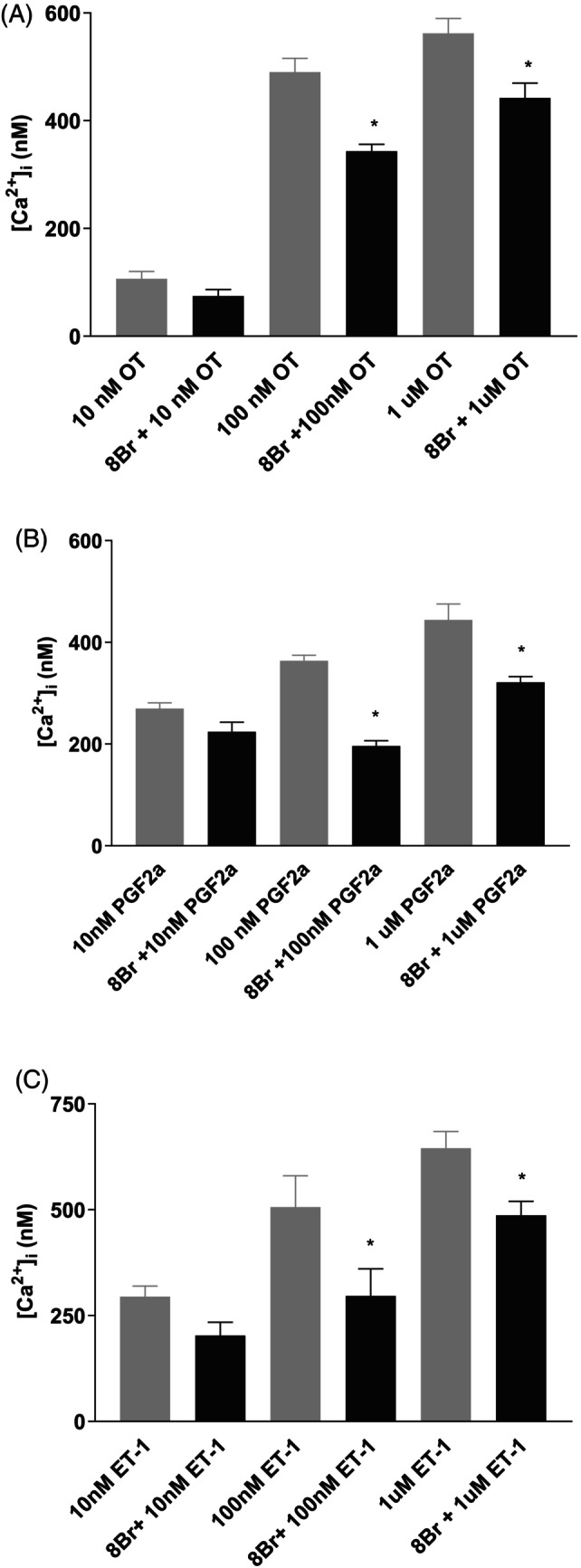
Agonists induced intracellular calcium response and the effects of 8‐Br‐cADPR on different concentrations of agonists induced [Ca^2+^]_i_ increase in PHM1 cells. The effects of 8‐Br‐cADPR on different concentrations of oxytocin (A), PGF2α (B), and ET‐1 (C) induced [Ca^2+^]_i_ elevation. Note, 8‐Br‐cADPR significantly (*p* < .05) inhibited 100 nM and 1 μM agonists induced the [Ca^2+^]_i_ increase while it had no significant effect (*p* > .05) on 10 nM agonists induced [Ca^2+^]_i_ increase in all three agonists. ET‐1, endotelin‐1; Oxy, oxytocin; PGF2α, prostaglandin F2alfa. Values are mean ± SEM. *Significantly different from its control without 8‐Br‐cADPR (*p* < .05).

Using digital video fluorescence imaging, we determined the [Ca^2+^]_i_ elevation in response to different concentrations of oxytocin, PGF_2α_, and ET‐1. Stimulation of PHM1 cells with the agonists resulted in a bi‐phasic pattern of elevation of [Ca^2+^]_i_ (Figure 3). The elevation of [Ca^2+^]_i_ in PHM1 cells was agonist concentration‐dependent. The net [Ca^2+^]_i_ elevation induced by 10 nM, 100 nM, and 1 μM of oxytocin were 107 ± 13, 490 ± 26, and 562 ± 27 nM, respectively (Figure 4A). Similarly, the net [Ca^2+^]_i_ elevation in response to 10 nM, 100 nM, and 1 μM of PGF_2α_ were 270 ± 11, 364 ± 10, and 444 ± 32 nM, respectively (Figure 4B). The [Ca^2+^]_i_ elevation upon ET‐1 stimulation was also concentration‐dependent in PHM1 cells. The net [Ca^2+^]_i_ elevation to 10 nM, 100 nM, and 1 μM of ET‐1 were 295 ± 25, 506 ± 74, and 645 ± 40 nM, respectively (Figure 4C).

### 3.4 Effect of 8‐Br‐cADPR on agonist‐induced intracellular Ca^2+^ increase in PHM1 cells

We determined the effect of 8‐Br‐cADPR, a membrane‐permeant cADPR antagonist on oxytocin, PGF2α, and ET‐1‐induced elevation in the [Ca2+]i in PHM1 cells. Pre‐incubation of PHM1 cells with 100 μM of 8‐Br‐cADPR significantly (*p* < .001) attenuated [Ca^2+^]_i_ elevation elicited by all three agonists, with significant inhibition of net [Ca^2+^]_i_ elevation in response to 100 nM and 1 μM of each of the agonists by 8‐Br‐cADPR. The net inhibition of [Ca^2+^]_i_ responses to 100 nM and 1 μM of oxytocin by 8‐Br‐cADPR were 30% and 21.4%, respectively (Figure 4A). Likewise, the net inhibition of [Ca^2+^]_i_ responses to 100 nM and 1 μM of PGF_2α_ by 8‐Br‐cADPR were 46.1% and 27.6%, respectively (Figure 4B). The net inhibition of [Ca^2+^]_i_ responses to 100 nM and 1 μM of ET‐1 by 8Br‐cADPR were 41.5% and 24.6%, respectively (Figure 4C).

**FIGURE 5:**
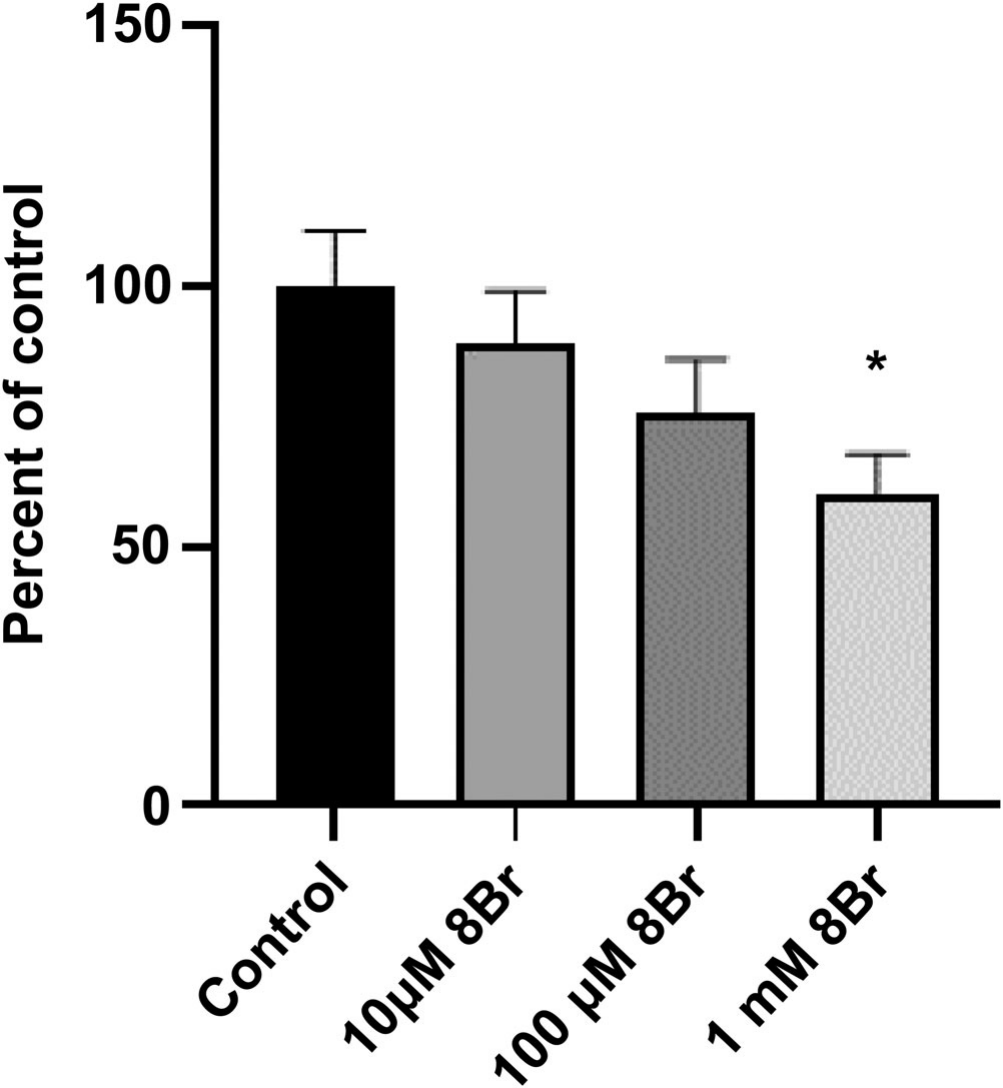
The concentration dependence of 8‐Br‐cADPR effect on oxytocin‐induced [Ca^2+^]_i_ response in PHM1 cells. The cells were pre‐incubated with different concentrations of 8‐Br‐cADPR for 15 min and subsequently perfused with 100 nM of oxytocin. Values are mean ± SEM. *Significantly different from control group (*p* < .05).

To examine the concentration dependence of 8‐Br‐cADPR, PHM1 cells were stimulated with 100 nM oxytocin. Pre‐incubation of PHM1 cells with 10 μM, 100 μM, and 1 mM of 8‐Br‐cADPR resulted in a significant (*p* < .05) inhibition of net [Ca^2+^]_i_ elevation to 100 nM oxytocin. The inhibition of net [Ca^2+^]_i_ elevation in the presence of 10 μM, 100 μM, and 1 mM of 8‐Br‐cADPR were 19%, 24%, and 40%, respectively (Figure 5).

## 4 DISCUSSION

In this study, we demonstrated CD38 mRNA and protein expression in the immortalized pregnant human myometrial (PHM1) cells. The expression of CD38 was associated with both ADP‐ribosyl cyclase and cADPR hydrolase activities, which are an integral part of CD38. Furthermore, uterine contractile agonists such as oxytocin, PGF_2α_, and ET‐1 increased [Ca^2+^]_i_ levels in PHM1 cells in a concentration‐dependent manner, and 8‐Br‐cADPR, a membrane‐permeant cADPR antagonist, attenuated agonist‐elicited increments of intracellular calcium levels.

A gestational human myometrial cell line was developed and has been used to study signal transduction mechanisms in the myometrium. The PHM1 cells maintained in culture have been well characterized, and previous studies have demonstrated that these cells maintain the morphological and biochemical properties of uterine smooth muscle cells.^25^, ^30^ Furthermore, it was demonstrated that these cells express functional receptors for oxytocin, relaxin, and estrogen, similar to primary uterine smooth muscle cells.^21^, ^26^, ^31^, ^32^ Moreover, transcriptome profiles of PHM1 cells were similar to primary human myometrial cells with an *r* = 0.9.^33^ Hence, PHM1 cells form a good model system to study signal transduction mechanisms and also to discover drug targets in the uterine smooth muscle cells, as an alternative to primary cells isolated from human uterine samples.

CD38 is a membrane‐bound protein and found to be expressed ubiquitously on different types of cells.^6^, ^7^, ^10^, ^11^, ^12^, ^13^, ^14^ The functional role of CD38 is predominantly due to the enzyme activities it possesses, namely ADP‐ribosyl cyclase and cADPR hydrolase.^2^ These enzymes are involved in the metabolism of cADPR, a calcium‐mobilizing second messenger. Its expression has been shown in vascular,^34^ intestinal,^35^ bladder,^36^ bronchial,^37^ tracheal,^15^, ^27^ and uterine smooth muscles.^17^, ^23^, ^38^, ^39^ Lee et al., showed different levels of CD38 protein and mRNA expression in different vascular smooth muscles.^34^ Sieck et al., also demonstrated that ASM (Airway Smooth Muscle) cells obtained from human bronchi exhibited significant increment both in the expression of CD38 upon TNF‐alpha treatment and ADP‐ribosyl cyclase activity. Furthermore, in Sieck's study, CD38 knockdown achieved by siRNA attenuated TNF‐alpha‐induced changes in ASM.^37^ Consistently, Deshpande et al., demonstrated that human ASM cells obtained from trachealis muscle exhibited significant enhancement in CD38 expression and ADP‐ribosyl cyclase activity with TNF‐alpha treatment. Furthermore, their response to calcium was also significantly augmented. The increment was attenuated by cADPR antagonist 8‐bromo‐cADPR.^27^ Earlier studies primarily used rodent models and relied on tissue preparations, demonstrating the expression of enzyme machinery involved in NAD metabolism and cADPR‐mediated calcium regulation in uterine contraction. Limited number of studies investigated the relevance of this signaling mechanism in human myometrial cells. This stems, in part, due to the lack of access to human cells. Our study utilized an immortalized cell line that retains signal transduction machinery involved in G protein‐coupled receptor signaling relevant to myometrial contraction. Thus, our study findings go beyond demonstrating the role of CD38‐cADPR in calcium regulation in myometrial smooth muscle cells, which will provide a basis for utilizing this as a model to study multiple signal transduction mechanisms. While cell culture models are valuable in signal transduction studies, they often are limited by the loss of expression of key enzymes or effector molecules. Thus, establishing the molecular identity of enzyme machinery, second messenger(s), and effector molecules in a particular pathway is critical in exploiting these models in future studies. As for myometrium, it was demonstrated that cADPR acts through RyRs in the myometrium and this effect could be abrogated by ryanodine and ruthenium red suggesting that cADPR‐mediated calcium release occurs through activation of RyR channels in the myometrium.^17^ In myometrial smooth muscle cells, both RYR1, primarily found in skeletal muscle, and RyR2, primarily found in cardiac muscles, are reported to be expressed. In this context, literature reported the expressions of all subtypes of ryanodine receptors (RyR1, RyR2, and RyR3) in myometrium.^40^ In addition, both, RyR1 and RyR2 have been reported to be more common in human pregnant myometrium.^41^, ^42^ On the contrary, some literature claims that contribution of RyRs to intracellular calcium release is minimal in mouse, rat, or human.^43^ Thus, cADPR‐induced Ca^2+^ release from RyR in myometrium could be from all three subtypes of RyRs. Moreover, using microsomes prepared from human myometrial cells, it has been demonstrated that cADPR induces calcium increase which was inhibited by 8‐Br‐cADPR, but not by xestospongin C, an IP_3_ inhibitor. These findings prove that cADPR modulates calcium release through RyRs.^17^ Furthermore, Thompson et al. reported TNF‐alpha treatment increased CD38 mRNA and protein expression levels with a 3‐fold increase in ADPR cyclase activity which was blocked with siRNA in primary human myometrial cells.^18^

In the present study, we demonstrated the expression of CD38 and the presence of ADP‐ribosyl cyclase and cADPR hydrolase activities in PHM1 cells. The findings are consistent with previous studies done with the primary rat and human uterine smooth muscle cells.^17^, ^23^ In the current study, a detectable level of cADPR was maintained in PHM1 cells and this level is comparable to the levels of cADPR in human myometrial cells and rat tissues. The findings suggest a possible functional role for the CD38/cADPR in PHM1 cells similar to primary uterine smooth muscle cells.

The roles of agonists such as oxytocin, PGF_2α_, and ET‐1 on uterine muscle contraction have been studied by many investigators.^39^, ^44^, ^45^, ^46^, ^47^, ^48^ These agonists modulate uterine muscle contraction by controlling intracellular calcium levels.^41^, ^44^ In the present study, we demonstrated that stimulation of PHM1 cells with agonists oxytocin, PGF_2a_, and ET‐1 results in the elevation of [Ca^2+^]_i_. These results are consistent with the previous report which used acutely dissociated primary human uterine smooth muscle cells.^17^ However, in the present study, we used multiple contractile agonists, and the findings suggest that CD38/cADPR‐mediated calcium signaling is a common pathway for multiple agonists. The findings of this article suggest that PHM1 cells possess the functional receptors for these contractile agonists similar to the primary uterine smooth muscle cells. Furthermore, we demonstrated that 8‐Br‐cADPR, a cell‐permeant cADPR antagonist, significantly inhibited the [Ca^2+^]_i_ responses induced by uterine contractile agonists suggesting a role for CD38/cADPR in the regulation of agonist‐induced intracellular calcium in the myometrial smooth muscle cells. However, it is important to note the studies using 8‐Br‐cADPR revealed that the net [Ca^2+^]_i_ elevation elicited by the lowest concentration of the agonists was not sensitive to inhibition. This may be explained by the fact that the [Ca^2+^]_i_ responses to 10 nM agonists may reflect an increase exclusively through IP_3_R channels, while at higher concentrations, the agonists may recruit both the IP_3_ and the RyR channels in the SR. It is also possible that another protein of the ADP‐ribosyl cyclase family could be responsible for the synthesis of cADPR as it is shown in the literatures.^49^

To our knowledge, the present study is the first one to identify CD38 mRNA and protein expression in gestational human myometrial cells. It is also the first to report cADPR agonist and antagonist‐induced intracellular calcium changes with CD38 activity in gestational human myometrial cells. In literature, it has been clearly denoted that hormonal regulation in gestation affects multiple receptor expression.^50^, ^51^, ^52^, ^53^ Consistently, our laboratory has previously demonstrated in term‐pregnant rats, CD38 mRNA and protein expression were higher compared with preterm ones, resulting in a significantly higher cADPR activity in term‐pregnant rats suggesting hormonal regulation of CD38 as well.^23^ Therefore, it is crucial to investigate agonist and antagonist‐based responses in gestational tissues to investigate further therapeutic effect, as agonists‐antagonists hold a highly substantial role in uterine contraction and labor. It should be mentioned that all these experiments were done at room temperature and agonists or antagonist may have different effects in working physiological conditions, especially in a condition like labor in a live organism. Taken together, the differentially regulated portion of a gestational tissue is crucial and is the essential part to investigate possible therapeutic agents that can be utilized in labor. With this motive, we utilized PHM1 cells to elucidate a gestational tissue response. We have characterized the presence of CD38/cADPR signaling machinery in PHM1 cells and investigated the contribution of cADPR‐mediated calcium increase to the overall intracellular calcium elevation induced by multiple contractile agents. The findings of the study may pave the way for the development of new drugs which target CD38/cADPR to modulate myometrial contraction during uterine pathologies. Findings will also establish PHM1 cells as one of the in vitro models to study the role of various signaling mechanisms in the physiology of uterine smooth muscle including the CD38/cADPR signaling pathway. We encourage further studies utilizing gestational tissues to reveal distinct metabolic properties—if any and to develop advanced therapeutics that can be used in labor.
